# Predicting the course of Alzheimer’s progression

**DOI:** 10.1186/s40708-019-0099-0

**Published:** 2019-06-28

**Authors:** Samuel Iddi, Dan Li, Paul S. Aisen, Michael S. Rafii, Wesley K. Thompson, Michael C. Donohue, for the Alzheimer’s Disease Neuroimaging Initiative

**Affiliations:** 1 Alzheimer’s Therapeutic Research Institute, Keck School of Medicine, University of Southern California, San Diego, USA; 2 Department of Family Medicine and Public Health, University of California, San Diego, USA; 3 Department of Statistics and Actuarial Science, University of Ghana, Legon-Accra, Ghana; 4 African Population and Health Research Center, APHRC Campus, Manga Close, Off Kirawa Road, P.O. Box 10787-00100, Nairobi, Kenya

**Keywords:** Alzheimer’s disease, Biomakers, Classification Clinical diagnosis, Disease trajectories, Joint mixed-effects models, Latent time shift, Model averaging, Multi-level Bayesian models, Multi-cohort longitudinal data, Predictions, Random forest

## Abstract

Alzheimer’s disease is the most common neurodegenerative disease and is characterized by the accumulation of amyloid-beta peptides leading to the formation of plaques and tau protein tangles in brain. These neuropathological features precede cognitive impairment and Alzheimer’s dementia by many years. To better understand and predict the course of disease from early-stage asymptomatic to late-stage dementia, it is critical to study the patterns of progression of multiple markers. In particular, we aim to predict the likely future course of progression for individuals given only a single observation of their markers. Improved individual-level prediction may lead to improved clinical care and clinical trials. We propose a two-stage approach to modeling and predicting measures of cognition, function, brain imaging, fluid biomarkers, and diagnosis of individuals using multiple domains simultaneously. In the first stage, joint (or multivariate) mixed-effects models are used to simultaneously model multiple markers over time. In the second stage, random forests are used to predict categorical diagnoses (cognitively normal, mild cognitive impairment, or dementia) from predictions of continuous markers based on the first-stage model. The combination of the two models allows one to leverage their key strengths in order to obtain improved accuracy. We characterize the predictive accuracy of this two-stage approach using data from the Alzheimer’s Disease Neuroimaging Initiative. The two-stage approach using a single joint mixed-effects model for all continuous outcomes yields better diagnostic classification accuracy compared to using separate univariate mixed-effects models for each of the continuous outcomes. Overall prediction accuracy above 80% was achieved over a period of 2.5 years. The results further indicate that overall accuracy is improved when markers from multiple assessment domains, such as cognition, function, and brain imaging, are used in the prediction algorithm as compared to the use of markers from a single domain only.

## Introduction

Prediction of future Alzheimer’s disease (AD)-related progression is extremely valuable in clinical practice and in medical research. In clinical practice, the ability to accurately predict the diagnosis of a patient can help physicians make more informed clinical decisions on treatment strategies [1]. Clinical trials are more likely to be successful if the individuals selected for the trials are those most likely to benefit from the therapy. Many researches in the field contend that preventative strategies initiated prior to the appearance of advanced symptoms are most likely to be successful [2–4]. Therefore identifying candidates for therapies while they are still cognitively normal (CN) or mildly cognitively impaired (MCI) is key for clinical trials, and eventually clinical practice.

The pathology of AD is characterized by the accumulation of amyloid plaques and neurofibrillary tangles in the brain beginning as early as middle age. The amyloid hypothesis posits that plaques caused by the gradual buildup of beta-amyloid (${\text {A}}\beta$) peptides damage brain regions responsible for cognition thereby leading to impairment. Recent studies have shown that the pathology of the disease occurs several years before the onset of clinical symptoms, making the disease difficult to detect at an early stage [5, 6]. In addition, prediction of the future diagnosis of an individual (CN, MCI, or dementia) is very challenging due to high subjectivity and individual-level variability in cognitive assessments and levels of biomarkers, which have typically been used for staging of AD. The assessment of an individual’s current diagnosis can vary from one clinician to the next, or from one day to the next.

Classification and prediction based on expert knowledge, machine learning algorithms [7, 8], regression-based prediction models [9, 10] and some combinations of these [11] have been proposed. Beheshti *e*t al[12] recently developed a computer-aided diagnosis system to predict conversion from MCI to AD using magnetic resonance imaging (MRI) data. Zheng *e*t al[13] surveyed other automated techniques for classifying and predicting diagnosis with reasonable reliability using data from different imaging modalities. The reliability of these approaches is often assessed by the sensitivity and specificity of the methods, accuracy rate, and absolute error rates, among other criteria. Approaches with high accuracy rates and precision are desirable. The diagnosis of CN, MCI, or mild dementia by expert clinicians has traditionally relied on cognitive assessments such as the Mini-Mental State Examination (MMSE) [14], Logical Memory [15] and structured clinical assessments such as the Clinical Dementia Rating (CDR) [16]. However, including multiple domains might help explain and more accurately predict the varying rates of decline that are typical. For example, it is common to find individuals who present with symptoms consistent with MCI or mild AD dementia, but who lack biomarker evidence of AD pathology. Such an individual might have other pathology that will exhibit a different rate of progression. Going beyond the cognitive domain to multi-domain analysis is therefore appealing. Longitudinal cognitive assessments combined with neuroimaging and biomarkers can more easily facilitate diagnosis and increase prediction accuracy [3, 17]. While multi-domain analyses are interesting, intuitive and potentially more informative, they have been relatively uncommon due to modeling challenges.

The Alzheimer’s Disease Prediction Of Longitudinal Evolution (TADPOLE) Challenge [18] is a challenge that compares performance of algorithms at making future predictions of AD disease markers and clinical diagnosis using historical data form the Alzheimer’s Disease Neuroimaging Initiative (ADNI) study. Motivated by this challenge, we aim to propose a two-stage approach that can reliably predict an individual’s future course of disease, including transition to MCI and dementia, using **only a single assessment** (i.e., “baseline”). This emphasis on subject-level prediction from a single timepoint is distinct from much of the literature which focuses on group-level prediction and the relative importance of various predictors. In the first stage, we model continuous disease markers using joint mixed-effects models.

In the first stage, the joint mixed-effect model allows the simultaneous modeling and prediction of multiple modalities such as cognitive and functional assessments, brain imaging, and biofluid assays with fixed effects for covariates like age, sex, and genetic risk. Joint models have the advantage of modeling the correlation among outcomes to improve prediction and precision of estimates [19, 20].

In the second stage of prediction, a random forest algorithm is used to categorize the panel of predicted continuous markers into a diagnosis of CN, MCI, or dementia. Random forests combine many decision trees created from random sampling of the data and predictors [21]. Each decision tree recursively partitions the predictors to classify individuals into one of the three diagnoses. While an alternative approach might view diagnosis as a random variable correlated with other disease markers, we view diagnosis as a deterministic categorization of the clinical presentation of each individual. That is, diagnosis should be algorithmically determined for given presentation of the continuous markers. The random forest model gives us an estimate of this algorithmic categorization. Overall performance is assessed using an independent validation set.

## Data description

The two-stage approach is applied to data from the Alzheimer’s Disease Neuroimaging Initiative (ADNI). ADNI is a prospective observational cohort study, which began in 2004 and continues to this day. The study is carried out across 55 research centers in the USA and Canada. Over 1900 volunteers with normal cognition or impairment consistent with MCI or AD dementia were recruited for this study. The first cohort, referred to as ADNI-1, consists of 800 individuals: 200 CN, 400 with late MCI, and 200 with mild dementia. ADNI-GO, the second cohort, added about 200 additional individuals with early MCI. In ADNI-2, more participants at different stages of AD were recruited to monitor AD progression. ADNI-3 is presently enrolling additional individuals with CN, MCI, and dementia. At each new phase, prior cohorts were invited back for continued follow-up, with the exception of individuals enrolled with dementia, who were followed for a maximum of 2 years. Some ADNI-1 individuals have now been followed in excess of 10 years. Key objectives of ADNI are to validate the use of markers of AD for diagnosis and clinical trials, and to study rates of change in cognitive and functional assessments, brain imaging and a number of biomarkers. The inclusion and exclusion criteria, schedule of assessments, and other details can be found at http://adni.loni.usc.edu/. We focus on the following assessments: Alzheimer’s Disease Assessment—Cognitive 13-item scale (ADAS13), Clinical Dementia Rating—Sum of Boxes (CDRSB), Mini-Mental State Examination (MMSE), Montreal Cognitive Assessment (MOCA), Rey Auditory Verbal Learning Test Immediate (RAVLT Immediate), Everyday Cognition (ECog)—total by participant (ECogPtTotal) and study partner (ECogSPTotal) and Functional Assessment Questionnaire (FAQ). Brain imaging measures include volumetric Magnetic Resonance Imaging (MRI) summaries of entorhinal cortical thickness, and ventricular and hippocampal volume normalized to intracranial volume (ICV); and fluorodeoxyglucose positron emission tomography (FDG-PET) summaries of glucose metabolism. Baseline diagnosis, age, gender, and carriage of APOE e4 allele were included as covariates.

We also focus on a second set of analyses among individuals where beta-amyloid data were available. The buildup of beta-amyloid in the brain and in cerebrospinal fluid (CSF) is known to be strongly involved in AD [22, 23]. For some patients in the ADNI study, florbetapir PET scans or CSF ${\text {A}}\beta 42$ was acquired to detect amyloid levels in brain. We classified individuals as having elevated amyloid (“amyloid positive”) if florbetapir PET standardized uptake value ratio (SUVR) was above 1.10 [22, 24] or if CSF ${\text {A}}\beta$ was less than 909.6 pg/ml; and as amyloid negative otherwise. The CSF ${\text {A}}\beta$ cutoff was determined so that it yielded the same proportion of amyloid positives as the florbetapir cutoff. Amyloid elevation status was included as a predictor in this second set of analysis.

## Methodology

We propose a two-stage approach for prediction of continuous disease markers and categorical diagnosis. For the first stage, we propose the traditional joint, or multivariate outcome, mixed-effects model; but we also consider two alternative approaches. We also consider a latent-time joint mixed-effects model and a Bayesian model averaging combining posterior estimates of the aforementioned joint models. In the second stage, the predicted markers are submitted to a random forest to further predict diagnosis. We next describe the first-stage model in greater detail.

### Methods for predicting continuous markers

Suppose $y_{ijk}$ represents *k* outcomes $(k=1, \ldots , p)$ observed at time $t_{ij}\ (j=1, \ldots , q_i)$ for each individual, $i \ (i=1, \ldots ,n)$, and ${\varvec{x}}_{ijk}$ is a set of covariates for the *i*th individual at time *j*. The joint mixed-effect model is defined

1 $$\begin{aligned} y_{ijk}={\varvec{x}}'_{ijk}{\varvec{\beta }}_k+ \alpha _{0ik}+\alpha _{1ik}t_{ij} +\varepsilon _{ijk} \end{aligned}$$

where ${\varvec{\beta }}_k; k=1,2, \ldots , p$, are sets of fixed-effect regression coefficients, $\alpha _{0ik}$ and $\alpha _{1ik}$ are outcome- and individual-specific random intercepts and slopes, respectively. The random intercepts and slopes are assumed to follow a multivariate normal distribution with mean vector, ${\varvec{0}}$ and variance–covariance matrix, ${\varvec{D}}$ for the entire 2*p*-dimensional vector of random effects for each subject. The error term follows $\varepsilon _{ijk}\sim N(0,\sigma ^2_k)$. The assumed homogeneity is over time of the error term for a given outcome and across all subjects. We assume that the random components ${\varvec{\alpha }}_{ik}$ and $\varepsilon _{ijk}$ for $k=1,2, \ldots , p$ are independent. The random effects allow the model to accommodate both the temporal correlation and correlation among the markers. A special case of this joint model is the independent mixed-effects model (IMM), which does not explicitly model the correlation among outcomes. This is similar to fitting separate mixed-effects model per outcome.

We also consider the latent time joint mixed-effects model (LTJMM) [25]:

2 $$\begin{aligned} y_{ijk}={\varvec{x}}'_{ijk}{\varvec{\beta }}_k+ \gamma _k (t_{ij}+\delta _i) +\alpha _{0ik}+\alpha _{1ik}t_{ij} +\varepsilon _{ijk}. \end{aligned}$$

The model is similar to 1, but introduces individual-specific latent time shifts, $\delta _i$, representing “long-term” disease time. The model also includes outcome-specific slopes $\gamma _k>0$ with respect to $\delta _i$. The $\delta _i$ are assumed to be normally distributed with zero mean and variance, $\sigma _{\delta }^2$. The random components, $\delta _i$, ${\varvec{\alpha }}_{ik}$ and $\varepsilon _{ijk}$ for $k=1,2, \ldots , p$ are also assumed to be independent. An extension of this model to allow heterogeneous latent-time (i.e., the variability of the latent-time is made to vary across individuals) is described in [26].

Estimation of the joint models is by Markov Chain Monte Carlo (MCMC). Posterior draws are obtained from the posterior distributions of the joint models given respectively by:

$$\begin{aligned} \begin{array}{rcl} P({\varvec{\theta }}|\mathbf{Y })&{}\propto &{} P(\mathbf{Y }|{\varvec{\theta }})P({\varvec{\theta }}|{\varvec{\tau }})\\ P({\varvec{\beta }}_k, {\varvec{\alpha }}_{i,k}|y_{ijk})&{}\propto &{} P(y_{ijk}|{\varvec{\beta }}_k, {\varvec{\alpha }}_{i,k},{\varvec{D}},\sigma ^2_{k}) P({\varvec{\beta }}_k)\\ &{}\times &{} P({\varvec{\alpha }}_{ik}|{\varvec{D}})P({\varvec{D}})P(\sigma ^2_{k}) \end{array} \end{aligned}$$

where the variance–covariance matrix, ${\varvec{D}}$ is decomposed as ${\varvec{D}}=\mathbf{V }{\varvec{\Omega }}\mathbf{V }$. For numerical stability, the Cholesky factorization is applied to the correlation matrix, ${\varvec{\Omega }}=\mathbf{L }\mathbf{L }'$, where $\mathbf{L }$ is a lower triangular matrix. For the latent time joint mixed-effects model, ${\varvec{\theta }}=({\varvec{\beta }}_k, {\varvec{\alpha }}_{i,k},\gamma _k,\delta _i)'$ and ${\varvec{\tau }}=({\varvec{D}}, \sigma ^2_{k})'$. The component, $\mathbf{V }$ is a diagonal matrix of standard deviations (square-root of diagonal entries of ${\varvec{D}}$). Furthermore, the random component, ${\varvec{\alpha }}_{ik}$ is standardized to $\mathbf{z }\sim N({\varvec{0}}, \mathbf{I })$, where $\mathbf{I }$ is the identity matrix and the random effects are then calculated as $\mathbf{V }\mathbf{L }\mathbf{z }$. Prior distributions are placed on the hyperparameters. A weakly informative normal prior, $N(0,10^2)$ is placed on ${\varvec{\beta }}_k$, and a weakly informative half-Cauchy prior, ${\text {Cauchy}}(0, 2.5)$, is assumed for the components of $\mathbf{V }, \sigma _{k}, \gamma _k$ and $\sigma _{\delta }$. Finally, the *LKJ* prior is placed on the Cholesky factors of ${\varvec{\Omega }}$ [27]. MCMC sampling is done using the R software package, RStan [28]. We used 5000 iterations, and the first 2500 warmup iterations are discarded. Two MCMC chains were used and thinned by a factor of 5. Predictions of biomarkers and their corresponding credible intervals were based on posterior draws. We apply Bayesian model averaging to the multivariate mixed models for the selected continuous biomarkers [29, 30]. The predictions of future values of biomarkers and the corresponding credible intervals are obtained after combining all posterior prediction estimates of all the models (model averaging). Suppose $y_{ijk}^*$ is the prediction of outcome *k* for individual *i* at future time *j*. The posterior distribution of the prediction given the data, *D* is the average of posterior distribution of the models weighted by the posterior model probabilities and is given by

$$ P(y_{ijk}^*|D)=  \sum _{s=1}^{S}P(y_{ijk}^*|M_s, D)P(M_s|D). $$

where $M_s, s=1,2, \ldots , S$ represents the models. The posterior distribution of the models is expressed as

$$ P(M_s|D)\propto  P(D|M_s)P(M_s) $$

where $P(D|M_s)=\int P(D|{\varvec{\theta }}_s, M_s)P({\varvec{\theta }}_s|M_s)d{\varvec{\theta }}_s$ and ${\varvec{\theta }}_s$ is the vector of parameters under model *s*. The predicted mean and variance are obtained from the posterior distribution of the predictions.

The JMM, and LTJMM were fit to training data described in Sect. 4. To demonstrate the benefit of joint modeling, single or independent mixed-effects (IMM) model were fit to the data for comparison. For the JMM and IMM models, age, gender, APOEe4, and baseline diagnosis were included as covariates. The latent-time models did not include baseline diagnosis since including this would make the model parameters uninterpretable due to the presence of the latent-time component (see [25] for details). Two common model selection criteria are applied, the widely applicable information criterion (WAIC) or the leave-one-out information criterion (LOOIC) [31]. Models with lower values of WAIC and LOOIC are preferred.

The models described above are fitted to the training dataset in order to make follow-up prediction for subjects in the test dataset. However, in fitting these models to the training data, we propose to include baseline data for subjects in the test data to allow for the estimation of random effects for these subject. The estimated outcome-specific random intercepts and slopes for each subject are required to make the subject-level predictions. The resulting follow-up predictions are then used as inputs in the random forest for the next stage of algorithmically predicting diagnosis status.

### Method for predicting clinical diagnosis

The random forest algorithm is an ensemble learning method for classification and regression. It operates by generating several classification or regression trees and aggregating them. Each tree in the forest is constructed using bootstrap samples of the data. The algorithm, implemented in the R package “randomForest” [30], is fitted to the training dataset using 100 trees. In particular, diagnosis which was re-evaluated at every visit by clinicians was used as the target feature for the random forest, and predicted follow-up continuous markers and baseline predictors of subjects as input features. Observation times are also included as a continuous predictor. A number of individuals had incomplete assessments at some study visits, which the random forest algorithm is not able to accommodate. To avoid discarding these incomplete visits entirely when fitting the random forest, we apply an imputation method, the “MissForest” algorithm [32], to impute the missing values. This algorithm, implemented in the R package “missForest”, imputes missing values for mixed-type data (e.g., continuous and categorical) using a nonparametric random forest methodology. The method can flexibly accommodate mixed-type outcomes, complex interactions and nonlinear relationships among variables. In addition, it does not require the specification of a parametric model or distributional assumptions. To determine variables which are important for predicting the response, we use the variable importance plot, which depicts the influence of each variable characterized by the mean decrease in node impurity (Gini Index [21]).

### Model performance metrics

To evaluate the quality of the predictions of the continuous markers, we use two performance metrics. The first metric, the mean absolute error (MAE), is calculated as

$$\begin{aligned} {{\,\mathrm{MAE}\,}}=\frac{1}{N}\sum _{i=1}^{N}|\hat{P_i}-P_i|, \end{aligned}$$

where *N* is the observation count, $\hat{P_i}$ represent the predicted or forecasted future values, and $P_i$ is the observed value of the marker for an individual *i* in the test data. The second metric, which takes confidence interval widths into account, is the weighted error score (WES). It is the weighted sum of the absolute difference between the predicted and actual values for each continuous marker in the test data at each time point. That is,

$$\begin{aligned} {{\,\mathrm{WES}\,}}=\frac{\sum _{i=1}^{N}\hat{C_i}|\hat{P_i}-P_i |}{\sum _{i=1}^{N}\hat{C_i}}, \end{aligned}$$

where the weights, $\hat{C_i}$, is the inverse of the width of the confidence interval of predicted estimates for each individual. High values of MAE and WES denote poor predictive performance of the model.

The diagnoses provided by site clinicians is used as the ‘gold standard’ in assessing the accuracy of the predictions of diagnosis from the random forest algorithm. Performance is assessed on the basis of the overall accuracy and balanced classification accuracy (BCA). Overall accuracy is defined as the percentage of correct predictions out of all the predictions made. This metric tends to work better for data with balanced classes (e.g., equal number of CN, MCI, or dementia) but can provide a misleading assessment of performance for data with imbalanced classes. To account for possible class imbalance, we also use the overall BCA. The balanced classification accuracy for class, $\ell =1,2, \ldots ,L$ is obtained from

$$\begin{aligned} {{\,\mathrm{BCA}\,}}_{\ell }=\frac{1}{2}\left[ \frac{TP_{\ell }}{TP_{\ell } +FN_{\ell }}+\frac{TN_{\ell }}{TN_{\ell }+FP_{\ell }}\right] , \end{aligned}$$

where $TP_{\ell }$ is the number of true positives, $FN_{\ell }$ is the number of false negatives, $TN_{\ell }$ is the number of true negatives, and $FP_{\ell }$ is the number of false positives. That is, for each class, $\ell$, TP is the number of cases that are correctly predicted by the model and $TN_{\ell }$ is the number of cases in class, $\ell$, which are incorrectly classified into any of the other classes. Similarly, $TN_{\ell }$ for class, $\ell$ represents the number of cases in the other classes correctly labeled as belonging to class, $\ell$, and $FP_{\ell }$ is the number of cases which actually belong to the other classes but are wrongly classified to class, $\ell$. These balanced accuracies are aggregated to obtain the overall BCA score as follows:

$$\begin{aligned} {{\,\mathrm{BCA}\,}}=\frac{1}{L}\sum _{\ell }^{L}{{\,\mathrm{BCA}\,}}_{\ell }. \end{aligned}$$

Higher value of overall accuracy or BCA is indicative of good performance.

## Application and model validation

### Descriptive statistics and data preparation

The ADNI data consist of 1737 individuals enrolled in ADNI-1, ADNI-GO and ADNI-2, 19.7% of whom have dementia, 30.1% are CN and 50.2% are MCI at baseline. About 44.9% are females, and 55.1% are males. All follow-up data on ADNI-1 and ADNI-GO participants who did not continue into the ADNI-2 phase, form part of the training dataset. In addition, baseline data from individuals in ADNI-2 are included in the training data to allow estimation of their random effects for individual-specific predictions. The training data consist of 273 ADs, 154 CNs and 414 MCIs. The validation dataset consisted of currently available longitudinal data for ADNI-2 (i.e., the ADNI-1 and ADNI-GO who continued into ADNI-2, and additional newly enrolled subjects). This validation data consist of 7.7% ADs, 41.2% CNs and 51.1% MCIs. Figure 7a, b, in “Appendix”, shows the number of individuals at each visit in the training and test sets, respectively. To impose a minimum standard for visit completion, time points where CDRSB was not observed are omitted from the analysis dataset. As expected, the number of observations decreases over time from baseline due to attrition and administrative censoring. Summary measures of baseline outcomes for each diagnosis group are presented in Table 1.

**Table 1:** Summary measures at baseline for raw and imputed data

Diagnosis category	Outcomes	Imputed data			Raw data		
		*n*	Mean	SE	*n*	Mean	SE
Dementia	ADAS13	342	29.91	0.43	330	29.87	0.44
	CDRSB	342	4.39	0.09	338	4.41	0.09
	EcogPtTotal	342	1.91	0.02	144	1.90	0.05
	EcogSPTotal	342	2.75	0.03	145	2.74	0.05
	MMSE	342	23.22	0.11	338	23.22	0.11
	MOCA	342	17.52	0.20	142	17.12	0.38
	RAVLT immediate	342	22.81	0.41	335	22.85	0.41
	FAQ	342	13.14	0.38	337	13.18	0.38
	FDG	342	1.07	0.01	242	1.07	0.01
	Hippocampus/ICV($\times \, 100$)	342	0.38	0.00	272	0.38	0.00
	Ventricles/ICV	342	0.03	0.00	315	0.03	0.00
	Entorhinal (mm)	342	2829.35	33.72	254	2819.26	42.54
MCI	ADAS13	872	16.53	0.23	862	16.53	0.23
	CDRSB	872	1.52	0.03	866	1.52	0.03
	EcogPtTotal	872	1.84	0.01	468	1.79	0.02
	EcogSPTotal	872	1.84	0.02	465	1.72	0.03
	MMSE	872	27.59	0.06	866	27.59	0.06
	MOCA	872	22.66	0.09	465	23.41	0.15
	RAVLT immediate	872	34.24	0.36	866	34.24	0.36
	FAQ	872	3.18	0.14	862	3.17	0.14
	FDG	872	1.23	0.00	665	1.25	0.01
	Hippocampus/ICV($\times \,100$)	872	0.44	0.00	737	0.44	0.00
	Ventricles/ICV	872	0.03	0.00	836	0.03	0.00
	Entorhinal (mm)	872	3497.43	24.18	733	3497.38	27.67
CN	ADAS13	523	9.24	0.19	520	9.24	0.19
	CDRSB	523	0.04	0.01	520	0.04	0.01
	EcogPtTotal	523	1.41	0.01	290	1.41	0.02
	EcogSPTotal	523	1.22	0.01	288	1.21	0.02
	FAQ	523	0.24	0.04	520	0.24	0.04
	MMSE	523	29.06	0.05	520	29.06	0.05
	MOCA	523	25.54	0.09	287	25.76	0.14
	RAVLT immediate	523	44.66	0.43	518	44.67	0.43
	FDG	523	1.31	0.00	391	1.31	0.01
	Hippocampus/ICV($\times \,100$)	523	0.49	0.00	471	0.49	0.00
	Ventricles/ICV	523	0.02	0.00	494	0.02	0.00
	Entorhinal (mm)	523	3828.36	26.57	468	3840.29	29.09

*ADAS13* Alzheimer’s Disease Assessment Scale, *CDRSB* Clinical Dementia Rating—Sum of Boxes, *EcogPtTotal* everyday cognition participant, *EcogSPTotal* everyday cognition study partner, *FAQ* Functional Assessment Questionnaire, *FDG* FluoroDeoxyGlucose, *MMSE* Mini-Mental State Examination, *MOCA* Montreal Cognitive Assessment, *RAVLT* Rey Auditory Verbal Learning Test, *ICV* intracranial volume, *CN* control, *MCI* mild cognitive impairment, *n* number of observations, SE standard error

Figure 8a depicts the individual observed trajectories per outcome and also shows the length of years of follow-up. Figure 8b shows the individual trajectories after missing values have been imputed. It can be seen that the imputation algorithm appears to generate plausible values of missing data. Before fitting the models to the data, the original values of the outcomes were transformed into percentiles using a weighted empirical cumulative distribution function so that all outcomes are on a common scale. The weights were constructed using the inverse of the proportion of disease category for each outcome. The predicted values on the transformed scale are then back transformed into the original scale.

**Fig. 1:**
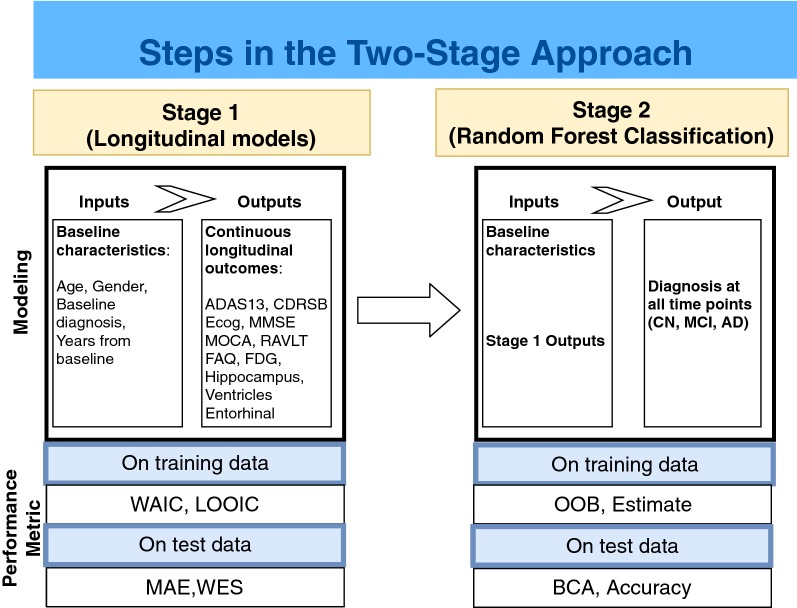
Schematic diagram showing the inputs and outputs of the two-stage approach

Next, we apply the two-stage approach to the data. Figure 1 shows a schematic diagram depicting the inputs and outputs at each modeling stage.

### Stage 1

**Fig. 2:**
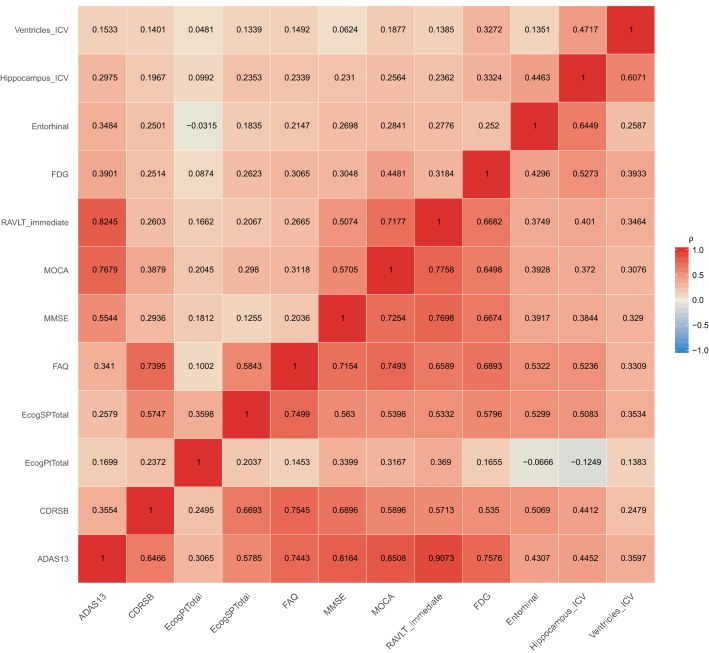
For each pair of outcomes, the correlations among random intercepts are above the anti-diagonal, and the correlations among random slopes are below the anti-diagonal. *ADAS13* Alzheimer’s Disease Assessment Scale,* CDRSB* Clinical Dementia Rating—Sum of Boxes,* EcogPtTotal* everyday cognition participant,* EcogSPTotal* everyday cognition study partner,* FAQ* Functional Assessment Questionnaire, *FDG* FluoroDeoxyGlucose,* MMSE* Mini-Mental State Examination,* MOCA* Montreal Cognitive Assessment,* RAVLT* Rey Auditory Verbal Learning Test,* ICV* intracranial volume

The joint mixed-effects models were trained on longitudinal data from ADNI-1, ADNI-GO, and only baseline data from ADNI-2. We then assessed the ability of the proposed methodology to accurately predict follow-up observations of individuals in ADNI-2. Table 2 summarizes WAIC and LOOIC. Based on these results, the JMM model seems to be the best fitting model, followed closely by the LTJMM model. Figure 2 shows the correlations between random intercepts (above anti-diagonal) and random slopes (below anti-diagonal) from the JMM. Cognitive outcomes share strong correlations [0.7–0.9) with other cognitive measures except for Everyday Cognition (ECog) by participant. There are generally moderate correlations [0.5–0.7) among cognitive measures and FDG-PET but weaker correlation [0.3–0.5) between cognition and structural MRI measures. There are generally moderate correlations among slopes for structural MRI measures.

**Table 2:** Model selection criteria

Model	WAIC	LOOIC
IMM	59,687.42	64,136.45
LTJMM	57,953.63	62,535.86
JMM	56,576.79	59,728.43

*WAIC* widely applicable information criterion, *LOOIC* leave-one-out information criterion, *IMM* independent mixed-effects model, *JMM* joint mixed-effects model, *LTJMM* latent-time joint mixed-effects model

**Fig. 3:**
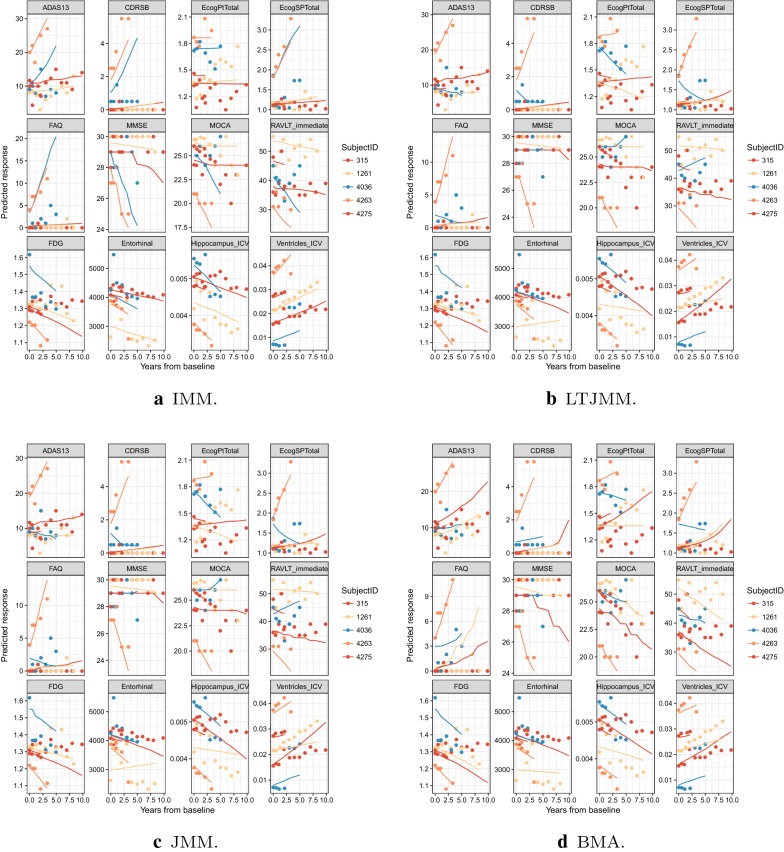
Observed values (points) versus predicted lines (lines) of markers for five randomly selected individuals for each of the four modeling approaches. *IMM* Independent mixed-effects model, *JMM* joint mixed-effects model, *LTJMM* latent-time joint mixed-effects model, *BMA* Bayesian model averaging, *ADAS13* Alzheimer’s Disease Assessment Scale, *CDRSB* Clinical Dementia Rating—Sum of Boxes, *EcogPtTotal* everyday cognition participant, *EcogSPTotal* everyday cognition study partner, *FAQ* Functional Assessment Questionnaire, *FDG* FluoroDeoxyGlucose, *MMSE* Mini-Mental State Examination, *MOCA* Montreal Cognitive Assessment, *RAVLT* Rey Auditory Verbal Learning Test, ICV intracranial volume

We also performed a Bayesian model averaging to combine predictions from the JMM, LTJMM and IMM. Furthermore, the joint mixed-effect model was fitted to cognitive and function outcomes (JMMCognitive), and imaging markers (JMMImage) to demonstrate how these marker domains perform individually. Longitudinal predictions on the validation dataset were obtained from these fitted models. Figure 3 shows the observed data and predicted trajectories for five randomly selected individuals for each model (in Fig. 9, we show plots for subject #315 and subject # 4263 where the models are all in the same panel, and subjects are in different panels for easy comparison). The graph shows that the models’ predicted profiles appear to differ only slightly. It is worth noting that, the predicted values appear nonlinear because the models were fitted to transformed values of the outcome and back transformed to the original scale.

**Fig. 4:**
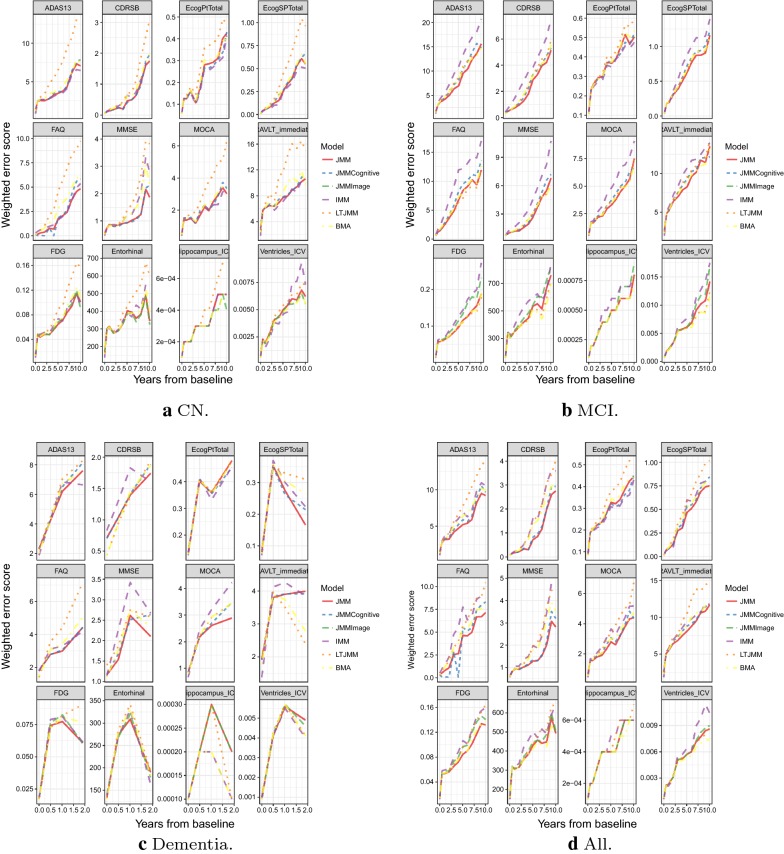
Validation set weighted error scores over time for each model by diagnosis. *CN* Control, *MCI* mild cognitive impairment, *IMM* independent mixed-effects model, *JMM* joint mixed-effects model, *LTJMM* latent-time joint mixed-effects model, *BMA* Bayesian model averaging, *JMMCognitive* JMM fitted to cognitive and function outcomes only, *JMMImage* JMM fitted to imaging markers only

We evaluated the performance of our model predictions using metrics on both the continuous markers and the multi-class diagnosis. The metrics described in Sect. 3.3 are used. From Figs. 10 and 4, we observed that predictions from all the joint models performed quite well over 2 years, yielding lower mean absolute errors and weighted error scores as compared to the other models. As expected, the MAE and WES increased beyond 2 years. All models yielded consistent performance over time with the JMMs occasionally out-performing the other models. The JMM that combined both cognitive and imaging outcomes performed similar to the JMM from cognitive/functional outcomes (JMMCognitive) and JMM from imaging markers (JMMImage) in terms of weighted error scores. However, at time points where the models differed, JMM with both cognitive and imaging outcomes was generally more accurate than JMMCognitive and JMMImage. The IMM performed worse for MCI and dementia subgroups.

### Stage 2

**Fig. 5:**
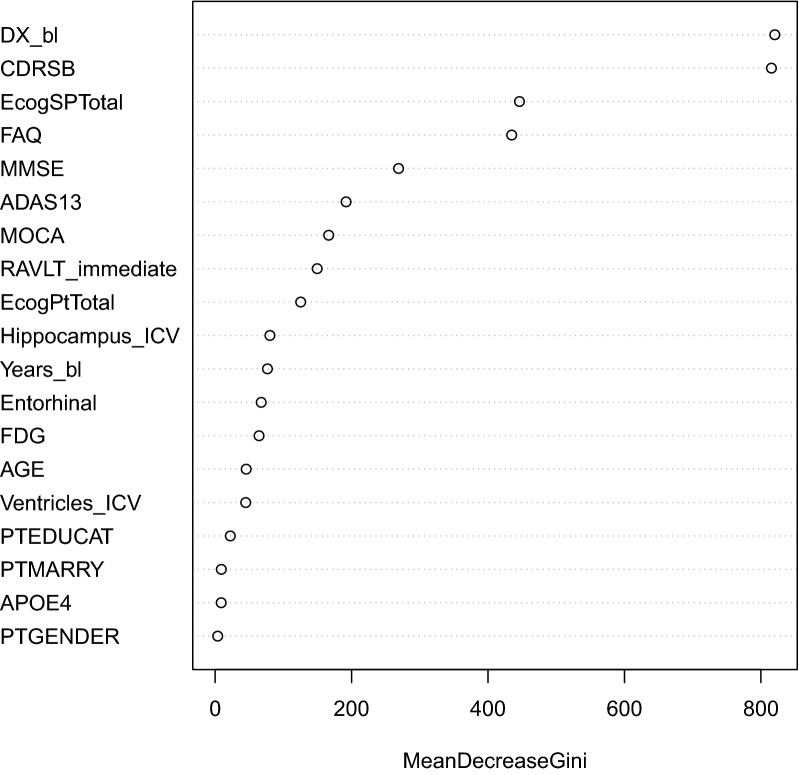
Random forest variable importance for categorical diagnosis (cognitively normal, mild cognitive impairment, or dementia). *ADAS13* Alzheimer’s Disease Assessment Scale, *CDRSB* Clinical Dementia Rating—Sum of Boxes, *EcogPtTotal* everyday cognition participant, *EcogSPTotal* everyday cognition study partner, *FAQ* Functional Assessment Questionnaire, *FDG* FluoroDeoxyGlucose, *MMSE* Mini-Mental State Examination, *MOCA* Montreal Cognitive Assessment, *RAVLT* Rey Auditory Verbal Learning Test, *ICV* intracranial volume, *PTGENDER* participant’s gender, *PTMARRY* participant’s marital status, *PTEDUCAT* participant’s education, *Year_bl* years from baseline, *DX_bl* baseline diagnosis, *APOE4* APOE e4 allele

**Fig. 6:**
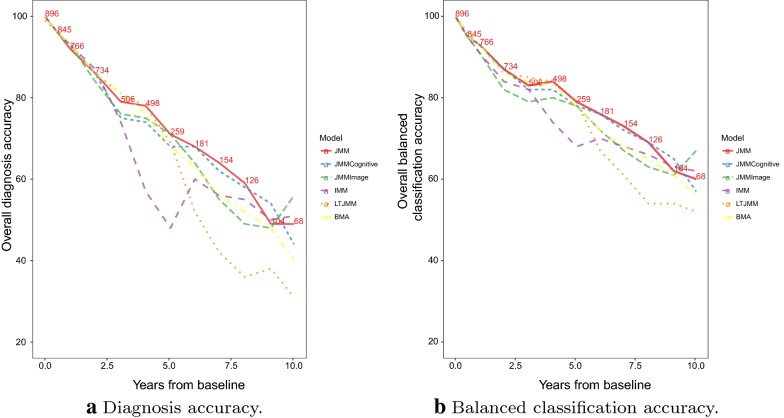
Comparison of performance metrics for categorical diagnosis. Note that only the LTJMM did not include baseline diagnosis as a covariate. The numbers on the graph represent the number of subjects at each of the time points. *IMM* Independent mixed-effects model, *JMM* joint mixed-effects model, *LTJMM* latent-time joint mixed-effects model, *BMA* Bayesian model averaging, *JMMCognitive* JMM fitted to cognitive and function outcomes only, *JMMImage* JMM fitted to imaging markers only

**Fig. 7:**
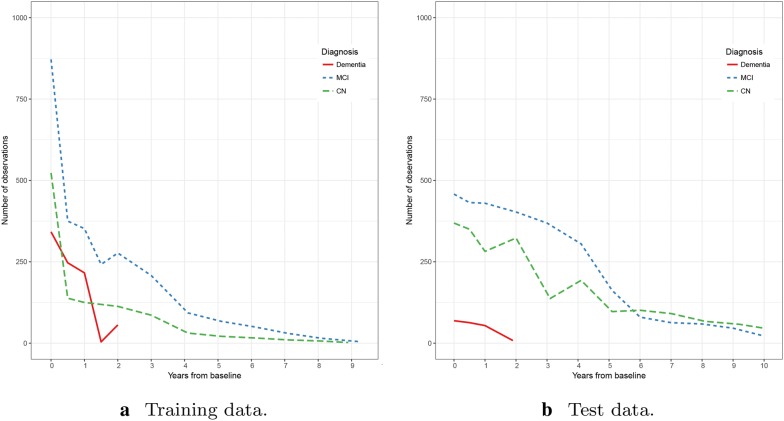
Number of individuals observed at each visit by initial diagnosis. *CN* Control, *MCI* mild cognitive impairment

**Fig. 8:**
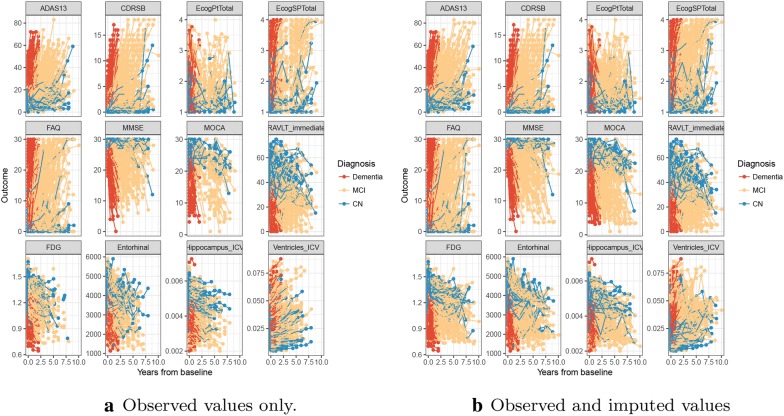
Observed and imputed values. The MissForest algorithm was used to impute missing values which appear to be plausible when compared to observed values at other visits. *CN* Control, *MCI* mild cognitive impairment

**Fig. 9:**
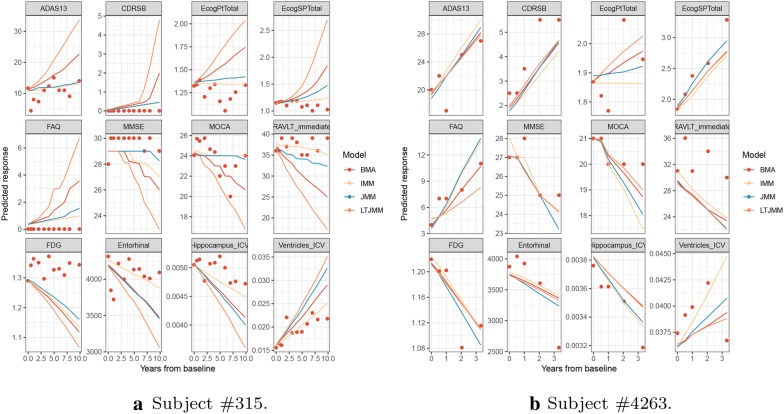
Observed values (points) versus predicted lines (lines) based on only their baseline data for each of the four modeling approaches for subject#314 and for subject#4263. *IMM* Independent mixed-effects model, *JMM* joint mixed-effects model, *LTJMM* latent-time joint mixed-effects model, *BMA* Bayesian model averaging, *ADAS13* Alzheimer’s Disease Assessment Scale, *CDRSB* Clinical Dementia Rating—Sum of Boxes, *EcogPtTotal* everyday cognition participant, *EcogSPTotal* everyday cognition study partner, *FAQ* Functional Assessment Questionnaire, *FDG* FluoroDeoxyGlucose, *MMSE* Mini-Mental State Examination, *MOCA* Montreal Cognitive Assessment, *RAVLT* Rey Auditory Verbal Learning Test, *ICV* intracranial volume

**Fig. 10:**
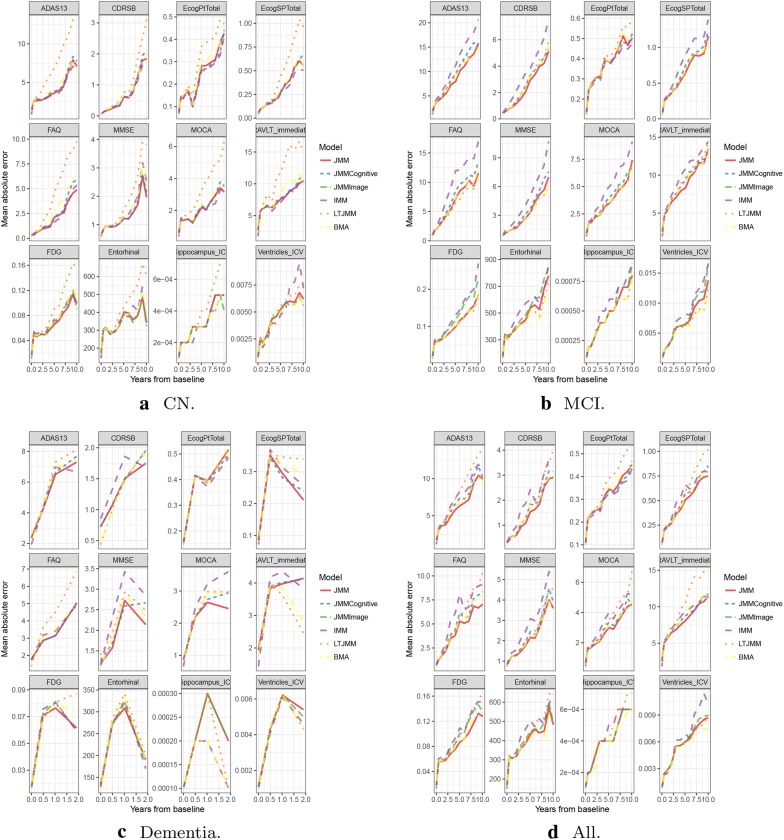
Mean absolute error. *CN* Control, *MCI* mild cognitive impairment

Table 3 shows the confusion matrix summarizing the within-sample classification accuracy of the random forest using observed continuous markers and baseline predictors in the training set. Predictors in the random forest classification algorithm included all continuous markers, years from baseline, and baseline characteristics such as age, education, marital status, APOE4 status and gender. An overall out-of-bag (OOB) estimated error rate of 4.55% was achieved. The variable importance plot in Fig. 5 shows the influence of each variable in predicting clinical status. The baseline diagnosis, CDR Sum of Boxes, Study Partner Everyday Cognition, Functional Assessment Questionnaire, and Mini-Mental State Examination are the features with the highest importance. The random forest predictions using predicted longitudinal markers from the joint models as inputs along with time-varying age, APOEe4 status and gender, achieve overall accuracy and balanced classification accuracy above 80% for periods less than 2 years (see Fig. 6). Between 2 and 5 years, we achieve an overall accuracy of between 60–80%. To facilitate overall comparisons, we computed BCA aggregated across all the time points and weighted according to the amount of data available at each time point. These weighted aggregate BCAs were 88.9%, 85.2%, 86.6%, 87.4%, 87.7% and 85.7% for JMM, IMM, LTJMM, BMA, JMMCognitive and JMMimage, respectively. This reinforces the interpretation that the JMM with both cognitive and imaging markers performs better than the models with either cognitive or imaging markers only.

### Sub-analysis for subjects with amyloid pathology information

**Fig. 11:**
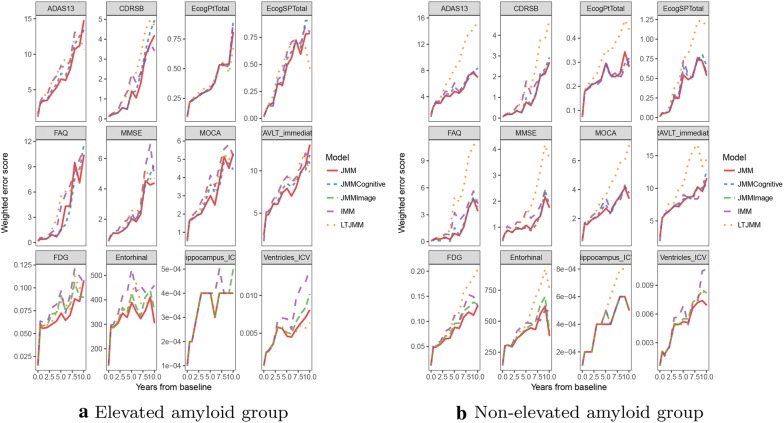
Weighted error score for subset of the population with amyloid burden information. *IMM* Independent mixed-effects model, *JMM* joint mixed-effects model, *LTJMM* latent-time joint mixed-effects model

**Fig. 12:**
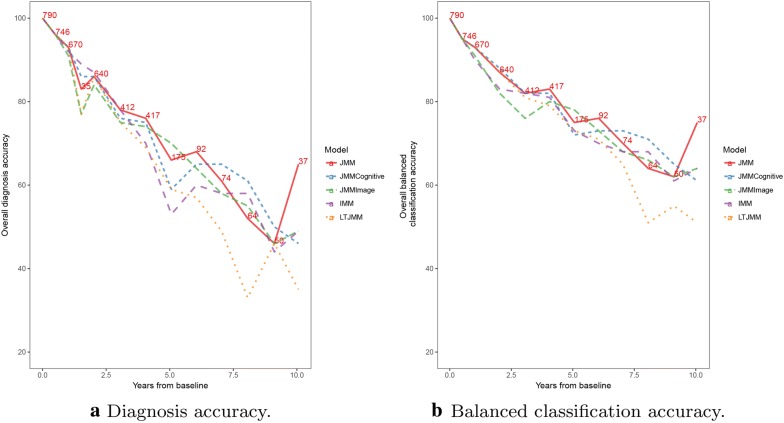
Comparison of performance metrics on clinical status for subset of the population with amyloid burden information. Note that only the LTJMM did not include baseline diagnosis as a covariate. The numbers on the graph represent the number of subjects at each of the occasions. *CN* Control, *MCI* mild cognitive impairment, *IMM* independent mixed-effects model, *JMM* joint mixed-effects model, *LTJMM* latent-time joint mixed-effects model

To explore the role of amyloid pathology, we applied our approach to a subset of the original data involving only individuals with amyloid information in both the training and test dataset as described in Sect. 2. Baseline amyloid elevation status was included as a predictor in both the random forest and multivariate mixed-effects models. To highlight the important role of amyloid status in the models, we compare the out-of-bag accuracy of the random forest with versus without including baseline amyloid status as a predictor on the subset of the training set with observed amyloid status. The OOB estimate of error rates were 4.99% and 5.13% for analysis with and without amyloid information, respectively. Thus, there is a modest added benefit with the inclusion of amyloid elevation status. This is not too surprising as the diagnostic classification in ADNI is based solely on the clinical presentation done without the clinicians’ knowledge of any biomarkers. Figure 11a, b shows the predictive performance of the continuous longitudinal markers under each of the joint models for groups of elevated and non-elevated amyloid individuals, respectively. We observed that the models predict follow-up biomarkers outcomes better for the individuals with non-elevated amyloid, owing to the fact that these individuals are likely to be more stable over time. The joint mixed-effects model continues to outperform the other models in terms of accuracy. Classification accuracy of clinical diagnosis is also depicted in Fig. 12. The random forest based on predictions from the joint models and baseline characteristics again yields balance classification accuracy of above 80% for the first two and a half years and declined over time. Again, the joint mixed-effects model combined with the random forest algorithm consistently outperformed the others.

**Table 3:** Confusion matrix

Actual	Predicted			Row total	Overall class error
	CN	MCI	Dementia		
CN	1218 (96.51%)	44 (3.49%)	0 (0.00%)	1262	0.035
MCI	29 (1.16%)	2382 (95.17%)	92 (3.68%)	2503	0.048
Dementia	0 (0.00%)	107 (4.84%)	2105 (95.16%)	2212	0.048
Column total	1247	2533	2197	5977	0.046

This confusion matrix summarizes the performance of the random forest algorithm for classifying diagnoses based on contemporaneous observations. The table compares actual diagnoses observed in the training set with diagnoses predicted by the random forest based on observed continuous data and baseline predictors

*CN* control, *MCI* mild cognitive impairment

## Discussion and conclusion

In this study, we have investigated the use of a two-stage data-driven approach to modeling and predicting the progression of AD markers and clinical diagnosis. Longitudinal data were jointly modeled to take advantage of correlations among outcomes and within individuals. Random forests were used to derive an algorithm to categorize diagnoses. Predictions were assessed on an independent validation set. The approach achieved overall accuracy and balanced classification accuracy of above 80% for the first 2 years, but accuracy diminished precipitously beyond 2 years. This finding supports the utility of our two-stage method for predicting disease course over a limited time frame. The findings also support the use of machine learning methods to derive algorithms which might help avoid subjectivity in diagnostic categorization.

A number of publications have addresses diagnostic prediction at various stages of AD. For example, Tierney et al. [33] attempted to predict the onset of dementia at 5 and 10 years based on an initial neurological test battery. By using a univariate logistic regression model, their approach yielded accuracies of 82% at 5 years and 71% at 10 years. Using a survival regression approach, Tabert et al. [34] predicted conversion from MCI to AD based on neurological batteries used as inputs and adjusted for other study participants’ characteristics. Their approach resulted in a 3-year predictive accuracy of 86%. Time-to-event outcomes generally have the ability to improve predictions over univariate logistic regression models. A more recent review by Rathore et al. [35] details how different classification frameworks have been used as an effective tool for making individualized diagnosis and prediction. Classification accuracies ranged from 70 to 95% for binary classification. These accuracies are impressive, but might not be comparable to the accuracies that we have reported. One reason for the incomparability is that the accuracies that we report are based on a held-out test that was not used to fit models. The accuracies we report also blend initial diagnoses and consider all possible transitions (multinomial outcome) of disease status rather than the binary approach adopted by these authors. For example, the classification approach by Tierney et al. [33] does not include MCI patients. However, it is generally more difficult to discriminate between adjacent diagnoses (e.g., cognitively normal and MCI) compared to non-adjacent diagnoses (e.g., cognitively normal and dementia).

The different approaches we considered for the “stage one” modeling each have their own strengths and weaknesses. The independent mixed model, for example, is easier to fit than the joint mixed-effects models and is also less cumbersome to interpret. However, this model ignores the correlations among outcomes which are generally known to be mild to strong for some pairs of AD markers. The correlation matrix of the random effects estimated in this study provides evidence of these between-outcome associations. On the other hand, joint models are complex, take more computational time, and can be challenging to interpret. In the presence of baseline diagnosis, the conventional joint mixed-effects model was preferred by the model selection criteria we considered. The latent-time joint mixed-effects model, motivated by the desire to predict long-term trajectories with short-term follow-up data, may be useful when baseline diagnosis is unknown. The Bayesian model averaging, which aggregates the other models, is probably the most complex but helps to account for model uncertainty in the estimation of parameters and prediction.

Some modifications might improve the prediction accuracy of the proposed two-stage algorithm. Instead of relying on a single time point to predict future course, one could utilize run-in data from multiple time points, which would likely improve estimates of subject-specific trajectories. Also, our models only considered a simple linear time trend. And while nonlinear trends were not supported by the data at hand, it is possible that a more flexible mean structure might improve model performance. Larger datasets and/or improved disease markers might also serve to enhance the quality of predictions in the future.

The approach can be applied to sharpen clinical trial inclusion and exclusion criteria to provide target populations with desired predicted longitudinal characteristics, e.g., a cognitively normal population with increased risk of imminent progression to MCI. However, such an application might complicate and prolong the recruitment process and eventual drug labeling.

In the clinic, these methods can be applied to improve the accuracy of prognosis. Improved prognostic accuracy can help physicians, patients, and families make more informed decisions regarding therapies and care through the transitions from healthy cognition, to mild impairment, to dementia. Once effective therapies have been discovered, the proposed two-stage approach could be fit to clinical trial data to provide a more sophisticated model of treatment response. Such a treatment response model, would provide personalized “theragnoses,” or predictions of treatment response; and help make decisions on when, and to whom, to prescribe therapies.

## Data Availability

ADNI data are disseminated by the Laboratory for Neuro Imaging at the University of Southern California. This work used the TADPOLE data sets https://tadpole.grand-challenge.org constructed by the EuroPOND consortium http://europond.eu funded by the European Union’s Horizon 2020 research and innovation programme under Grant Agreement No. 666992.

## Appendix: Supplementary appendix

See Figs. 7, 8, 9, 10, 11 and 12.

## Abbreviations

AD: Alzheimer’s disease
ADAS13: Alzheimer’s Disease Assessment—Cognitive 13-item scale
ADNI: Alzheimer’s Disease Neuroimaging Initiative
*APOE*: apolipoprotein E gene
BCA: balanced classification accuracy
BMA: Bayesian model averaging
CN: cognitively normal
CDRSB: Clinical Dementia Rating—Sum of Boxes
CSF: cerebrospinal fluid
ECog: everyday cognition
ECogPtTotal: ECog participant total
ECogSPTotal: ECog study partner total
FAQ: Functional Assessment Questionnaire
FDG: fluorodeoxyglucose
ICV: intracranial volume
IMM: independent mixed-effects model
JMM: joint mixed-effects model
JMMCognitive: JMM fitted to cognitive and function outcomes only
JMMImage: JMM fitted to imaging markers only
LTJMM: latent time joint mixed-effects model
LOOIC: leave-one-out information criterion
MAE: mean absolute error
MCMC: Markov Chain Monte Carlo
MMSE: Mini-Mental State Examination
MOCA: Montreal Cognitive Assessment
MRI: magnetic resonance imaging
PET: positron emission tomography
RAVLT Immediate: Rey Auditory Verbal Learning Test Immediate
SUVR: standardized uptake value ratio
WAIC: widely applicable information criterion
WES: weighted error score
